# Copper oxide nanoparticles fabricated by green chemistry using *Tribulus terrestris* seed natural extract-photocatalyst and green electrodes for energy storage device

**DOI:** 10.1038/s41598-023-49706-w

**Published:** 2023-12-15

**Authors:** Jayaprakash Meena, N. Kumaraguru, N. Sami veerappa, Paik-kyun Shin, Jiro Tatsugi, Annamalai Senthil Kumar, Kannappan Santhakumar

**Affiliations:** 1 Nano and Bioelectrochemistry Research Laboratory, Carbon Dioxide Research and Green Technology Centre, Vellore Institute of Technology, Vellore, 632 014 India; 2 Department of Chemistry, School of Advanced Sciences, Vellore Institute of Technology, Vellore, 632 014 India; 3 Department of Chemistry, Thanthai Periyar Government Arts and Science College, Tiruchirappalli, 620 023 India; 4 Department of Education, Government College of Education for Women, Coimbatore, 641 001 India; 5 School of Electrical Engineering, Inha University, Incheon, South Korea; 6 Department of Applied Chemistry, Aichi Institute of Technology, Toyota, Japan

**Keywords:** Environmental sciences, Chemistry, Materials science

## Abstract

Nanobiotechnology is a unique class of multiphase and recently become a branch of contemporary science and a paradigm shift in material research. One of the two main problems facing the field of nanomaterial synthesis is the discovery of new natural resources for the biological production of metal nanoparticles and the absence of knowledge about the chemical composition of bio-source required for synthesis and the chemical process or mechanism behind the production of metal nanoparticles presents the second difficulty. We reported template-free green synthesized copper oxide nanoparticles using *Tribulus terrestris* seed natural extract without any isolation process. XRD, TEM, SEM, UV–Vis, DLS, zeta potential, and BET evaluated the synthesized metal nanoparticle. The TEM analysis confirmed that the CuO NPs are well dispersed and almost round in shape with an average size of 58 nm. EDAX confirms that copper is the prominent metal present in the nanomaterial. The greener fabricated copper oxide nanoparticle was employed to degrade methyl orange dye, almost 84% of methyl orange was degraded within 120 min. The outcomes demonstrated the nanomaterial’s effective breakdown of contaminants, highlighting their potential for environmental rehabilitation. The electrochemical investigation of the CuO NPs was utilized for supercapacitor application. An appreciable value of specific capacitance is 369 F/g specific capacitances with 96.4% capacitance retention after 6000 cycles. Overall, the results of the current study show that the biologically produced copper oxide nanoparticles have intriguing uses as photocatalysts for treating water contaminants and are suitable for energy storage devices.

## Introduction

As a result, a tremendous amount of undesired man-made trash is dumped into the environment, causing serious environmental issues like pollution and causing severe ecological balance ^1,2^. So, nowadays researchers are looking to find new materials for a better environment. The basic criteria of the new material should satisfy multiple efficacies, with the properties of non-toxic, and environmentally safe and the preparation method should be eco-friendly and simple ^3^. The green synthesis method is one of the eco-friendly, safer methods due to the lessened usage of harsh chemicals ^4–6^. Plant-derived nanomaterials are a faster, straightforward, economical saver, and non-toxic technique than other methods. Intriguingly, the phytochemicals found in plant extracts—“terpenoids, alkaloids, phenols, tannins, flavonoids, saponins, secondary metabolites”, etc. would act as natural surfactants, reducing agents, capping agents, and stabilizing agents during the synthesis ^1,3,7–9^. There have been numerous reports of biogenic synthesis of different metal/metal oxide nanoparticles for various applications like energy storage, photocatalyst, medical engineering, drug delivery, sensing and tissue engineering, etc^7,10,11^ due to the high conductivity, large surface-to-volume ratio, plasmonic properties and so forth. The next generation of advanced technologies such as green nanotechnology, advanced materials and composites, and nanostructures for environmental remediation, wastewater treatment, renewable and clean energy production, and storage, addressing environmental issues, and electrochemical applications. Fabricating heterogeneous metal composites and engineered nanostructures has received more attention due to the good chemical stability in aqueous solution, desirable optical properties, and electrochemical properties. In the case of composites like SrTiO_3_^12^, Nd_2_Sn_2_O_7_^13^and CoFe_2_O_4_@SiO_2_@Dy_2_Ce_2_O_7_^14^ for photocatalytic applications, and CeVO_4_/rGO ^15^ and Nd_2_Sn_2_O_7_^16^ Zn_3_V_3_O_8_^17^ for hydrogen storage and Pr_2_Ce_2_O_7_^18^ and Pr_6_O_11_^19^ for electrochemical quantifications were synthesized. The other side is simple metal nanoparticles (Cu, Ni, Mn, Mg, V, Pt, Pd, Fe, Sn, and Ag), among all copper nanomaterials, have found appropriate applications due to their non-toxicity, availability, affordability, and ease of fabrication in the form of nanoscale materials. The current work focuses on the bioinspired synthesis of copper oxide nanoparticles (CuO NPs) utilizing the seed extract of *Tribulus terrestris* as a green reducer and surfactant in the biogenic synthesis of CuO NPs.

For the production of copper oxide NPs, various techniques have been employed by different researchers for different purposes. These consist of wet electrodeposition^20–22^ and chemical deposition^23,24^, chemical reduction techniques (hydrothermal)^19–22^, and others. These techniques require high pressure and temperature, and the majority employ hazardous chemicals that are harmful to people and the environment ^25,26^. Moreover, different plant extracts have been used to synthesize CuO NPs namely, *Moringa oleifera* plant extract ^27^, *Ficus benghalensis* leaf extract ^28^, *Clerodendrum phlomidis* extract ^29^, sugarcane molasses ^30^, *mimosa* leaves extract ^31^ for energy storage applications. *Commelina benghalensis* leaf extract ^32^, *Carica papaya* extract ^33^, and lemon peel ^34^ for dye degradation. Furthermore, apart from common metal nanoparticles like Zn, Au, Ag, Pt, and Ni. The new composites and advanced nanostructures were also tried for photocatalytic applications namely the facial synthesis of Pr_2_Ce_2_O_7_^35^, Nd_2_Sn_2_O_7_^13^, and Dy_2_O_3_–SiO_2_^36^ ZrO_2_^37^, and Sn–Ln mixed-metal oxides^38^ to enhance the material properties for pollutant degradation. Preparation of advanced materials composites, and nanostructures via a simple and green method and investigation of their performances is another domain of material research for specific applications.

In this work, we used natural extracts from fresh *T. terrestris* seed to synthesize simple CuO NPs and studied their potential for electrochemical energy storage application and photocatalyst. These days, dangerous chemicals like organic dyes are the main source of contaminated water. Organic dyes are widely utilized as colorants in a variety of industries, including printing, textiles, paper, food, medicine, and cosmetics. Numerous industrial effluents and organic dyes are frequently dumped into water bodies without adequate treatment. The high toxicity, carcinogenicity, and non-biodegradability of these dyes and their derivatives lead to a host of issues, including skin disorders, liver and kidney failure, and poisoning of living organisms' neurological systems. Developing an economical and environmentally friendly substitute for the breakdown of organic dyes in wastewater is crucial as the existing techniques for eliminating harmful materials from water bodies are both expensive and ineffective ^19,26,28^.

**Figure 1:**
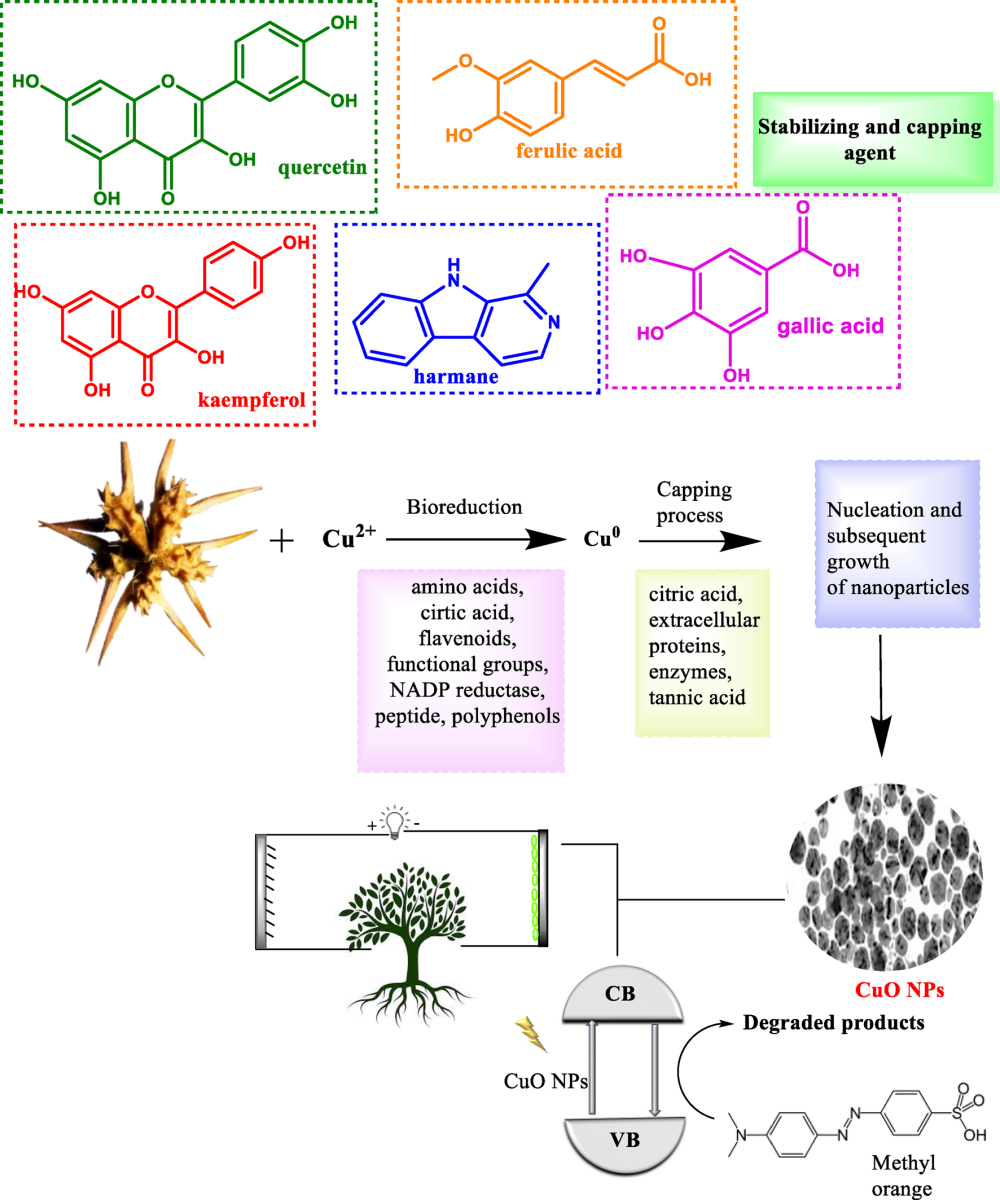
Bio-synthesis of CuO NPs for photocatalyst and supercapacitor application.

On the other hand, Due to their intermittent nature, energy storage is one of the main challenges faced by the majority of renewable energy sources, including wind and solar power ^39–41^. Over the past few decades, a variety of electrochemical energy storage devices, including fuel cells, various types of batteries, supercapacitors, and hybrid systems, have been developed ^41,42^. Among all supercapacitors have quick charge and discharge, long cycle life, and higher power densities than batteries. They can be used in various devices, including power electronic devices, solar cells, and biomedical sensors. Bioresources and biowaste materials can also be utilized to create supercapacitor electrodes ^43–45^. Two types of SCs are distinguished by their mechanisms of charge storage: the electric double-layer capacitor (EDLC) and the Faradaic reversible redox reaction of the pseudocapacitor (PC). In comparison to other conventional capacitors and the EDLC, the PC has a high specific capacitance and energy density. As a result, recent research has concentrated on creating PC electrodes to improve the performance of SCs. Because of their low cost, earth abundance, multi-oxidation states, high conductivity, and good stability, transition metal oxides have been regarded as active PC materials. When compared to pure metals and their oxides, metal-doped metal oxide nanostructures were specifically mentioned as promising pseudocapacitive electrodes. Prior studies have unequivocally shown that Ca doped with CuO electrodes improves electrochemical SCs' endurance, capacitance, surface area, and conductivity ^42^. As a result, the novelty of this work combines the benefits of employing sustainable resources with those of green chemistry concepts. In addition, synthesized CuO NPs have been observed to satisfy the important parameters of ecological material for dual roles such as photocatalysts used to degrade methyl orange and green electrodes for energy storage applications with good results. and (Fig. 1). In short, the findings of our research work may be considered serious, talented, and novel, suggesting that copper oxide synthesis via green synthesis might be engaged as a cost-effective and efficient adsorbent for pollutant adsorption and has wonderful potential for wastewater treatment and has the ability to store the energy for future energy devices—“Green material for a greener environment (dye degradation) and green energy (supercapacitor)”.

## Method and materials

### Chemicals list

We utilized analytical grade (AR) chemicals: Copper sulfate, *N*-*N*-Methyl pyrrolidone, PVDF, Nickel foam, KOH, and methyl orange were purchased from Sigma Aldrich and Merck companies and used as purchased, and no further purifications.

### *Tribulus terrestris* assisted synthesis of CuO

The CuO-NPs were synthesized by a traditional bio-reduction process by *T. terrestris* seed collected from Javadhu Hills, Eastern Ghats, Tamil Nadu, India. The appropriate permissions for collecting plant specimens were obtained from the administration affiliated with the Government of Tamil Nadu, India. Since it is a wild species flowers have been collected for our research and field study under the permission of competent authority of the State Forest Department, Govt. of Tamil Nadu. According to IUCN’s Red List Categories and Criteria, globally *T. terrestris* comes under the Least Concern (LC) category. We confirm that the collection of plant material complied with relevant institutional, national, and international guidelines and legislation.

20 g of *T. terrestris* seed was ground to form a powder by using mortar and pestle. ~ 2 g of *T. terrestris* seed powder was added into 50 ml of 0.2 M CuSO_4_ solution, stirred for 10 min, and heated in the hot plate at 60 °C for 1 h. The formation of CuO–NPs can be confirmed by color transformation to dark brown. The prepared solution was decanted and washed residue to remove gross by 50% alcohol and H_2_O followed by drying at 80 °C in a hot air oven.

### Material characterizations

High-resolution transmission electron microscopy (HR-TEM) equipped with Energy-dispersive X-ray spectroscopy (EDS) was performed in FEI TECNAI T20G2. Field emission scanning electron microscopy with Energy-dispersive X-ray spectroscopy was carried out in SU 8000, Hitachi, Tokyo, Japan. X-ray Diffraction (XRD) patterns were recorded at room temperature by JEOL JDX 8030. X-ray photoelectron spectroscopy (XPS) was carried out at Axis ultra DLD spectrometer (Kratos Co., UK) with a monochromatic Al-Kα X-ray source (1486.6 eV). The surface phenomenon was studied by atomic force microscope (INNOVA, USA), and the surface area analysis by Gemini VII 2.00. UV–Visible spectroscopy (UV–Vis) was carried out in JASCO V-560 and the photoluminescence by LABRAM (HR800) to study the optical properties of the extract and synthesized CuO-NP with diffuse reflectance. Fourier transforms infrared (FT-IR) spectroscopy was carried out in JASCO 440 plus. TGA-50 HIMADZU recorded thermogravimetric analysis. The zeta potential of the synthesized CuO NPs was analyzed by a 90-plus particle size analyzer (DR-525, Brookhaven Instruments Corporation, USA).

### Electrochemical characterization

All electrochemical characterizations were analyzed in CHI660E, USA. For electrochemical measurements, Ni foam is a working electrode, Platinum wire is the auxiliary electrode, and Ag/AgCl is the reference electrode. 0.5 ml of *N*-*N*-Methyl pyrrolidone (NMP), the solution was mixed with 1 mg of bio-synthesized CuO–NPs, and 10% PVDF binder and made as a paste. The slurry paste was painted on the pre-cleaned Ni foam (1 × 1 cm) and dried for 2 h at 25 °C and 3 M KOH solution as an electrolyte. The CV experiment was tested between the potential window of 0–0.65 V at various scan rates. The GCD curves were analyzed in the voltage range of 0 to 0.6 V at different current densities. Furthermore, EIS in the frequency of 0.001 kHz with a 5 mV amplitude was analyzed. The specific capacitances were calculated using a formula (1).

1 $${\text{Cs}} = i\Delta t/m\Delta v$$

whereas *i* = constant current, *m* = mass of active materials (mg) on the electrode surface, ∆*t* = galvanostatic discharge time in sec, and ∆*v* = applied potential window in volts.

### Photocatalytic activity of CuO NPs

The synthesized CuO NPs were used as a catalyst against methylene orange. A Heber multi-lamp photoreactor HML MP 88 with medium-pressure mercury vapor lamps (8W) was used for irradiation. As much as 100 mL of methylene orange solution (2.0 × 10^–5^ M) was mixed in a beaker containing 100 mg of synthesized CuO NPs. During the experiment, the mixture was continuously stirred and samples were collected every hour. UV–Vis spectrophotometer was used to observe the variation in dye absorption within the range of the 400–800 nm range. The outcome of the experiment was expressed as the percentage of degradation efficiency. Degradation efficiency (%) = initial absorbance(C_0_)- final absorbance(C)/initial absorbance(C_0_) X 100. Also, the obtained results are compared with recently synthesized CuO NPs by various methods.

### Mechanism of bioreduction

The major constitution of *T. terrestris seed* is kaempferol, astragalin, tribuloside, kaempferol 3-rutinoside, rutin, quercetin, harmane, coumaroyl-tyramine, ferulic acid, caffeoyl-tyramine, feruloyl-octopamine, coumaroyl-quinic acid ^46^. Those phytonutrients play an important role in the Bioreduction process and will act as capping agents to reduce metal ions into nanometal oxides (Mechanism 1)^9,47^

$${\text{Cu}}^{2 + } \left( {{\text{from CuSO}}_{4} \cdot 5{\text{H}}_{2} {\text{O}}} \right) + {\text{Phytonutrient }}\left( {{\text{from}}\;Tribulus\;Terrestris\;{\text{extract}}} \right) \to \left[ {\text{Cu - phytonutrient}} \right]^{2}$$

$$\left[ {\text{Cu - phytonutrient}} \right]^{2} \mathop{\longrightarrow}\limits_{{1{\text{H}}}}^{{60\;^{ \circ } {\text{C}}}}\left[ {{\text{Cu}}\left( {{\text{OH}}} \right)_{2} - {\text{Phytonutrient}}} \right] \mathop{\longrightarrow}\limits_{{{\text{Dried}}\;{\text{over}}\;{\text{night}}}}^{{{\text{Filtrate}}}}\left[ {\text{CuO Nps}} \right]$$

**Mechanism 1:**
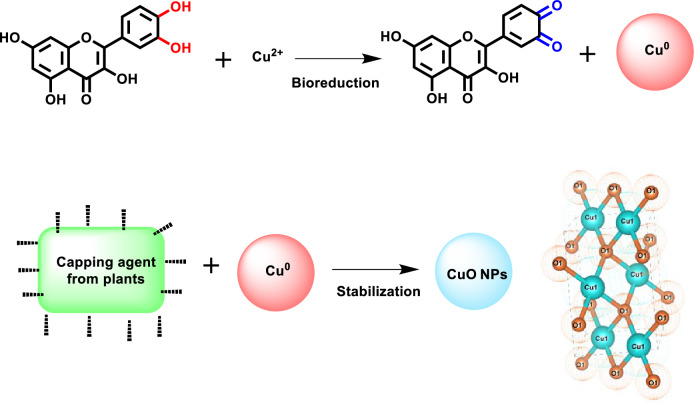
Mechanism of bioreduction processes using plant extract to synthesize CuO NPs.

## Results and discussion

### Exploring optical properties of Biosynthesized CuO NPs

#### UV–Vis spectroscopy

**Figure 2:**
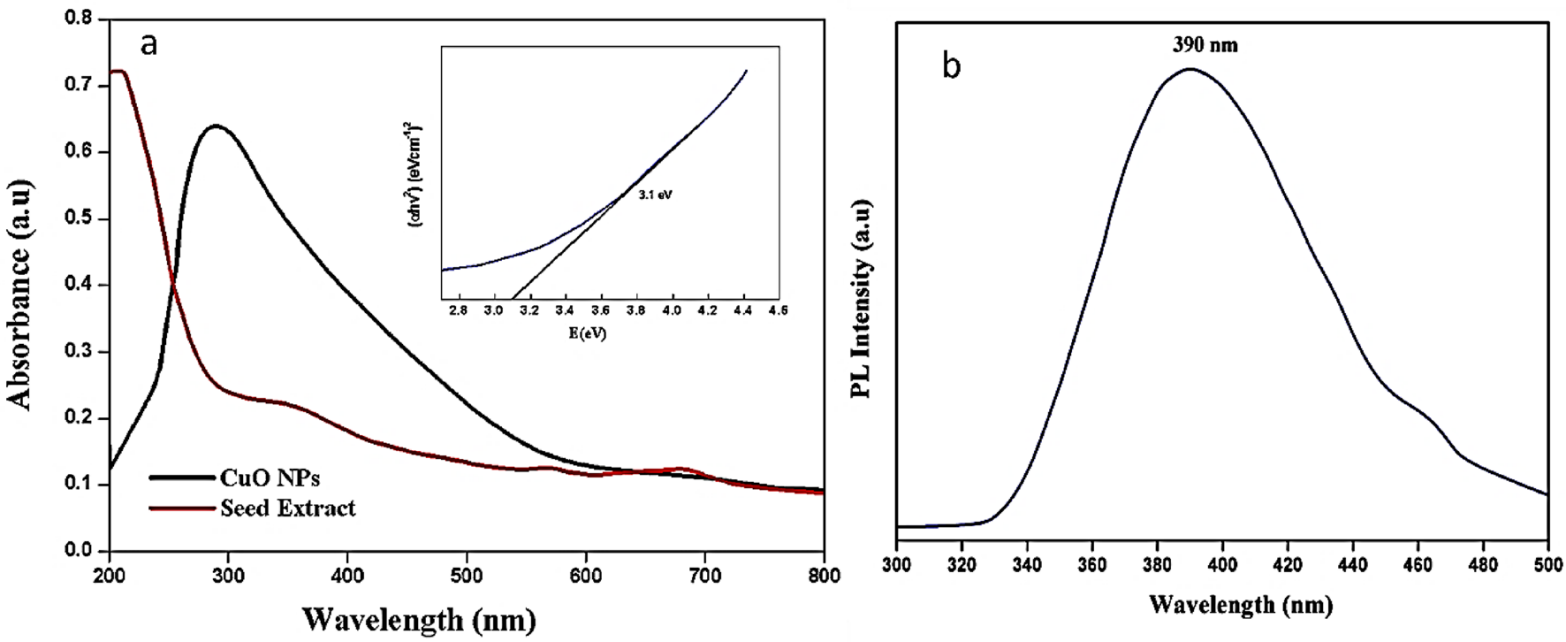
Biosynthesized CuO NPs (**a**) UV–Vis and (**b**) PL response.

The optical property of the synthesized CuO-NPs and seed extract is shown in Fig. 2a. The copper salt complexes are formed when the ions are reduced to form nanoparticles which were caused by the phytonutrients in the seed extracts. We utilized UV–Vis spectroscopy to analyze the color change in the produced solutions within the 200–800 nm range. Because of CuO NPs’ core electrons’ interband transition, For the production of CuO NPs, the broad peak center at 290 nm was observed and showed a drastic change from the *T. terrestris* seed extract. A significant absorbance peak between 250 and 300 nm was found by Sankar et al.^33^, which suggests the production of CuO NPs and the bandgap of 3.1 eV obtained from the Tauc’s plot.

#### Photoluminescence

The PL spectra dictate the important parameters of electronic energy transfer in nanoparticles and it varies based on the size and shape of the NPs, and additionally the excitation wavelength determination of the emission peak. At the excitation level of 390 nm and RT, PL spectra of as-synthesized CuO NPs were depicted in Fig. 2b. Normally, CuO is a p-type semiconductor, and the peaks vary due to the oxygen holes, surface defects, interstitial ion effect, and electron recombination between the donor and acceptor region of the nanoparticle. CuO Nps exhibited broad violet luminescence at 390 nm and the emission peaks were found at 390 nm and a feeble peak at 475 nm due to the band-edge emission and artifact ^48^.

### Exploring surface properties and structural composition of Biosynthesized CuO NPs

#### FT-IR spectrum of CuONPs and seed extract

**Figure 3:**
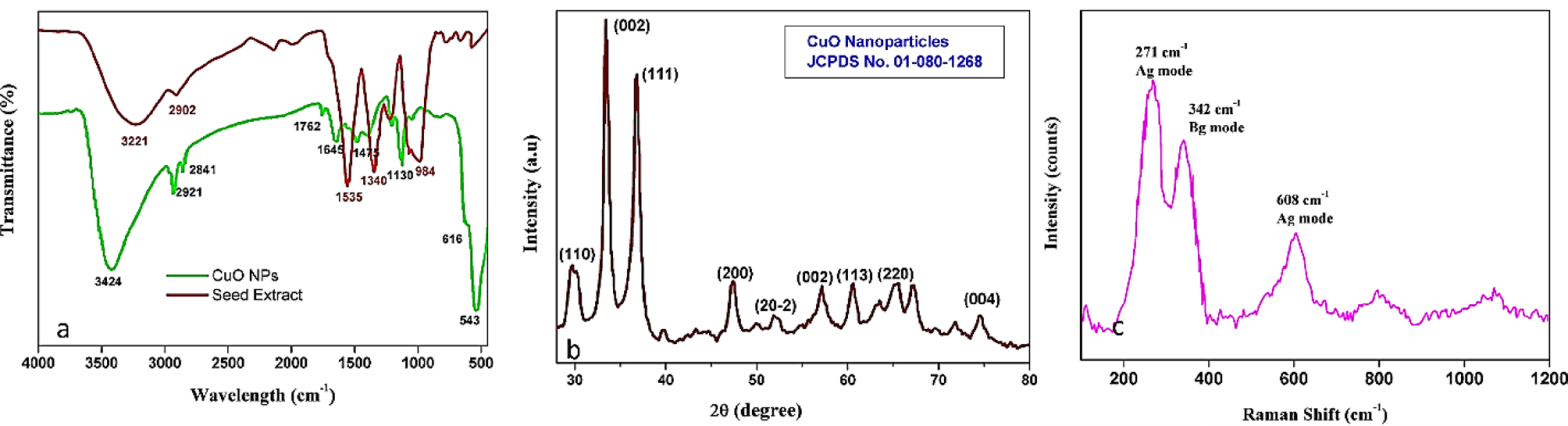
Biosynthesized CuO NPs (**a**) FT-IR (**b**) XRD and (**c**) Raman spectra.

FT-IR spectra of synthesized CuO–NPs and the *T. terrestris* seed extract are depicted in Fig. 3a. The Cuo–NPs and *T. terrestris* seed extract show the different functional groups due to the bio-reduction process of CuO and the presence of flavonoids, and polyphenols in *T. terrestris* seeds. The bands at 3424 and 3221 cm^−1^ can be assigned as O–H stretching in the carboxylic moiety (–COOH) and the hydroxyl group. The peak of 2921, 2841, and 2902 cm^−1^ shows the C–H stretching of alkane, 1762 and 1645 cm^−1^ resembles weak C=O stretching compared with seed extract has strong stretching vibrations at 1535, 1340, and 984 cm^−1^ represents N–H, C–N, C=C due to the polyphenols, flavonoids, terpenoids, and tannic acid and fails to show intense M–O bond whereas, the synthesized CuO-NPs shows the fine high intense band at 616, 543 cm^−1^ due to the formation of Cu–O bond^34,49^ with *T. terrestris* seeds extract, this confirms the formation of copper oxide nanoparticle and Bioreduction process.

#### XRD of CuO NPs

The crystalline phase and structural information of *T. terrestris-assisted* CuO NPs were investigated by the XRD patterns as shown in Fig. 3b. The interplanar spacing (d) for the corresponding peaks can be calculated using Brass’s law, nλ = 2dsinθ. The XRD diffraction peaks of CuO at 2θ = 29.89°, 33.56°, 36.97°, 47.57°, 51.88°, 57.44°, 60.85, 65.35, and 74.61° assigned as (110), (002), (111), (200), (202), (002), (113) and (004)^25,50^. The peaks of the CuO NPs (monoclinic phase) match JCPDS card No. 01-080-1268 with an average crystallite size of 58.7 nm calculated using Debye–Scherrer’s equation.

#### Raman spectra

Previous analysis has demonstrated that CuO with a monoclinic structure has a space group symmetry of C2h6 and that the Raman active is attributed to three zone-center optical phonon modes of Ag and 2Bg. The three peaks, 271 cm^−1^, 342 cm^−1^, and 608 cm^−1^, respectively, correspond to the A_g_, B_g_(1), and B_g_(2) modes of the copper oxide single crystal (Fig. 3c). The Raman active's peaks have widened and happened downshift in comparison to the Raman spectra of the CuO single crystal. The quantum confinement effect of the CuO nanoparticles is primarily responsible for the broadenings and blue shifts of the Raman peaks. CuO has 4 atoms in its primitive cell that give rise to 12 phonon branches. These consist of 3 acoustic modes (A_u_ + 2B_u_) and 9 zone-center optical phonon modes (4A_u_ + 5B_u_ + A_g_ + 2B_g_). However, only 3 symmetry modes (A_g_ + 2B_g_) are Raman active. They are positioned at the peaks of 298 (A_g_), 345 (A_g_), and 632 cm^−1^ (A_g_). The remaining 6 are IR-active (3A_u_ + 3B_u_). Only the oxygen atoms shift in the A_g_ and B_g_ Raman modes. They shift perpendicular to the b-axis in the case of the B_g_ modes. During the infrared active modes, both the O and Cu atoms are in motion. For the Au modes, the induced dipole moment is parallel to the b-axis. But for the Bu modes, it is perpendicular^49,50^.

#### XPS

**Figure 4:**
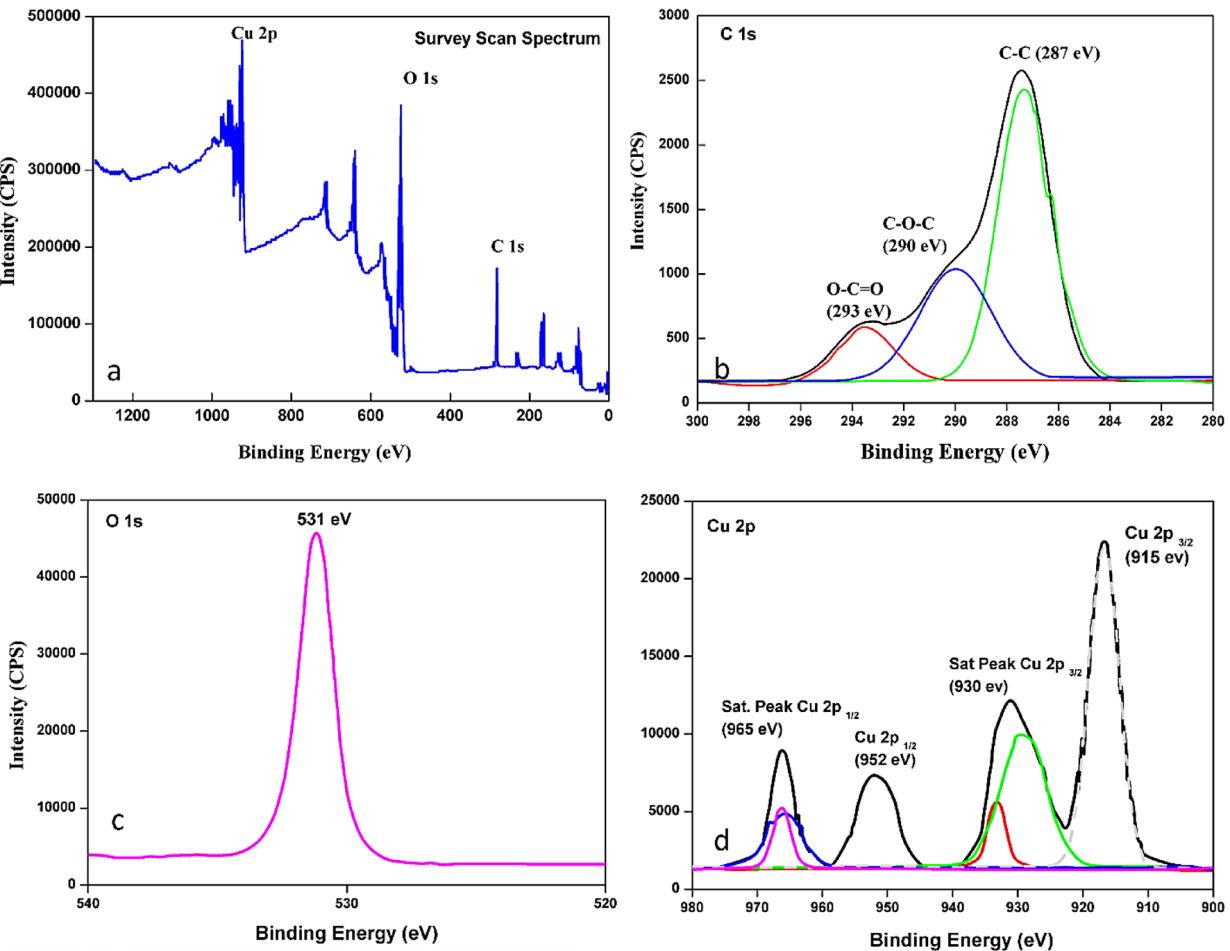
XPS response of biosynthesized CuO NPs (**a**) survey scan and elements of (**b**) C1s (**c**) O1s and (**d**) Cu 2p.

XPS is the preferable technique to analyze the chemical composition and electronic nature of the synthesized material by distinction on binding energies Fig. 4. shows the survey spectrum of CuO and the elements of Cu, C, and O were noticed. The Survey spectra (Fig. 4a) of CuO exhibit binding energy peaks corresponding to carbon (Fig. 4b), oxygen (Fig. 4c), and copper (Fig. 4d). By deconvoluting the Carbon peak into three distinct peaks, we identified C–C, C–O–C, and C=O bonds present in the *T.* *terrestris* Seeds, supported by evidence from FT-IR analysis. The appearance of C–O–C and C=O peaks at 290 eV and 293 eV, respectively, suggests the presence of hydroxyl and carboxyl groups from the seed extract. In the high-resolution XPS spectrum of the Cu 2p region, two peaks located at binding energies of 915 eV and 952 eV, were found as Cu 2p_3/2_ and Cu 2p_1/2_, respectively. Additionally, two shake-up satellite peaks were observed at binding energies of 930 and 965 eV, providing evidence of an open 3d9 shell characteristic of the Cu^+^ state. Furthermore, the (Fig. 4c) O1s binding energy at 531 eV closely resembles that of O^2−^ in CuO, confirming the formation of metal–oxygen (M–O) bonds in CuO. Overall, the XPS results validate the presence of specific chemical bonds and provide essential insights into the composition and electronic states within the CuO material ^25,49,51^.

### Exploring morphology investigation of Biosynthesized CuO NPs

#### Zeta potential

**Figure 5:**
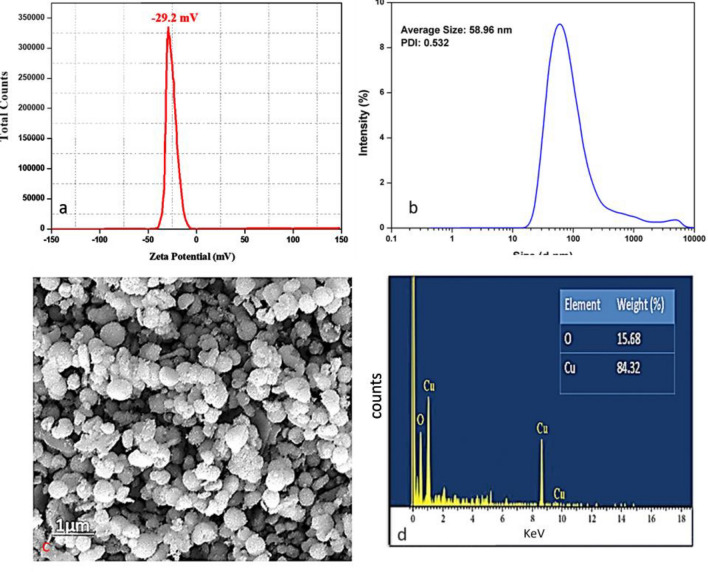
Biosynthesized CuO NPs response of (**a**) Zeta potential (**b**) DLS (**c**) SEM and (**d**) EDAX.

The zeta potential of *T. terrestris-assisted* CuO NPs indicates the degree of repulsion between opposite, same-charged particles in the dispersion in terms of the magnitude of charges and is found to be − 29.2 mV (Fig. 5a) it demonstrates quite a stability of CuO NPs at ambient temperature. A solution or dispersion can resist aggregation when the absolute value of the particle zeta potential in the solution is high. If attraction prevails over repulsion due to low zeta potential absolute values of the particles in a solution, the dispersion breaks down and flocculates. It is commonly accepted that a threshold of either + 30 or 30 mV marks the basic boundary between stable and unstable suspensions. Typically, Stable particles have zeta potentials of at least + 30 mV, primarily based on fabricated nanomaterials^52^.

#### Dynamic light scattering

DLS analysis was carried out to calculate the particle size (Fig. 5b). The CuO NPs created in the bio-reduction process utilizing plant leaf extract were widely distributed in the solution, according to the particle size analysis, and the particles made from the solution were substantially disseminated in the mixture, with sizes of 58.96 nm. The DLS approach, which measures the hydrodynamic radius, highly coincides with the XRD calculated size. Furthermore, the Polydispersity Index was found to be 0.532 which suggests that CuO–NPs are also highly monodispersed^49^.

#### SEM, TEM, and EDAX analysis of synthesized CuO NPs

**Figure 6:**
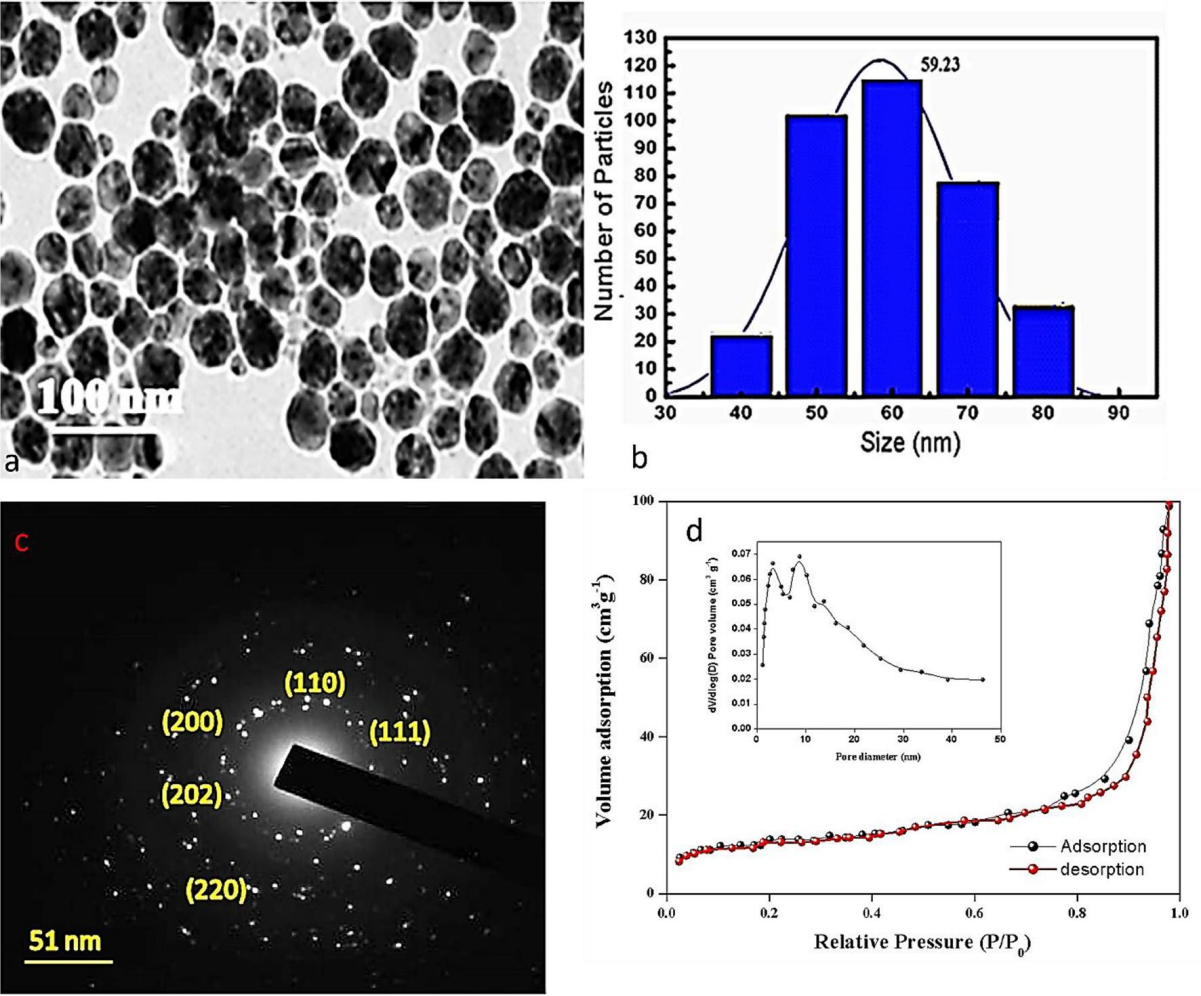
TEM images (**a**–**c**) and BET analysis (**d**) of synthesized CuO NPs.

To visualize the synthesized CuO NPs morphology and size, TEM (Fig. 6a,b) and SEM images were captured under an ambient atmosphere as shown in the figures it is clear from the image the synthesized CuONPs are spherical with an average size of ~ 59 nm and coincide with XRD crystalline size. From the SEM image (Fig. 5c, the accumulation in the synthesized CuONPs discloses diverse dimensions. Furthermore, elemental conformation was confirmed by EDX (Fig. 5d) merged with SEM to track the elements in synthesized NPs, the weight and atomic percentage of the elements present are shown in the EDS spectra of the copper oxide nanoparticles. The peaks of copper (84.3%) and oxygen (15.6%) are dominating in the absence of other contaminants. Eventually, the peak at 8.0 keV, dictates the formation of CuONPs. There were no other gross in the synthesized CuONPs. The presence of intermediate points within the concentration circles observed in the CuO nanoparticles, as revealed by the selected area electron diffraction (SAED), suggests that the nanoparticles are crystalline. Furthermore, the pattern closely matches the XRD pattern depicted in Fig. 6c, providing additional evidence of the nanoparticles' crystallinity.

#### BET

The CuONPs sample was used for the BET test to measure pore size, surface area, and pore volume. An illustration of a type IV isotherm nature with the H-3 hysteresis loop according to IUPAC classification, Fig. 6d (pore size distribution is inset) showed that CuONPs have a mesoporous structure. According to the BET data, the CuONPs created in this experiment have substantially smaller pores and more surface area than was expected. Additionally, the produced CuONPs' N_2_ adsorption isotherm displayed a BET surface specific area (SSA) of 32 m^2^/g, a pore size is 3–50 nm and a pore volume is 0.137 cm^−3^/g.

### Photocatalytic activity of MO

**Figure 7:**
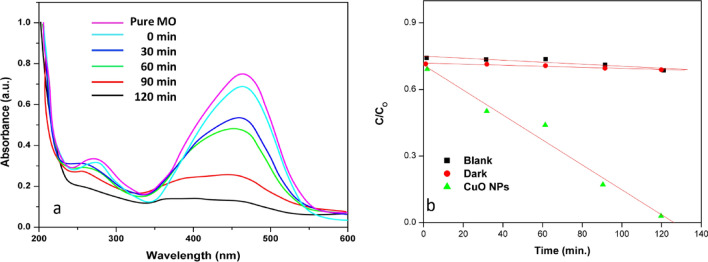
Photocatalyst (CuO NPs) response against methyl orange degradation with time as followed by UV–visible spectroscopy.

The photocatalytic experiment was conducted under three conditions: without the photocatalyst under light (Blank), with the photocatalyst in the dark (Dark), and with the photocatalyst under direct light. In this study, a concentration of 10 mg/L of methyl orange (MO) was selected as a model pollutant. It was found that the MO solution had a pH of 7.01. A Heber Multi lamp photoreactor HML MP 88 with medium-pressure mercury vapor lamps (8 W) was used for irradiation. In the experiment, we added 100 mg of CuO nanoparticles, synthesized using biological methods, to a 100 ml solution of methyl orange dye. Methyl orange dye has a chromophoric group, which gives it a maximum absorption at 464 nm. As shown in Fig. 6a, the dye's absorption reduces with increasing exposure to UV light, reaching a minimum after 120 min. The degradation efficiency is obtained 84.23% by using a photocatalyst. Here, we can see the cleavage of the chromophoric group (Mechanism 3) ^53,54^. Deamination from MO causes a blue shift from 464 to 440 nm as shown in Fig. 7a. Figure 7b shows a plot of dye concentration (C/C_O_) over time. A photocatalytic reaction (Mechanism 2a and b) produced sufficient hydroxyl radicals to degrade 84.23% of MO under UV light after 120 min. To confirm the efficiency of mineralization, COD values were used to evaluate the degradation part. We measured COD values before and after the photocatalytic treatment. A 120-min UV illumination significantly reduced COD values to 350 mg/l from 5020 mg/l. The degradation efficiency of bio-synthesized CuO nanoparticles is confirmed by the decreasing value of COD. Table 1 compares the various methods synthesized CuONPs for dye degradation of MO and Table 2 discusses the photodegradation efficiency of MO solutions on CuO nanoparticles obtained from different reaction conditions. TOF is one of the empirical parameters for the catalytic performance and is calculated using the below formula (2) and found to be TOF = 20.256 µ/min^55,56^.

2 $${\text{TOF}} = \left( {{\text{conversion }}\% } \right) \, \left( {{\text{No}}.{\text{ of moles of substrate}}} \right)/\left( {{\text{No}}.{\text{ of moles of catalyst }} \times {\text{ time}}} \right)$$

3 $${\text{CuO}} + {\text{MO}} \to \left[ {{\text{MO}} - {\text{CuO}}} \right]$$

4 $$\left[ {{\text{CuO}}} \right] + h\nu \to {\text{CuO}}\left( {{\text{e}}^{ - }_{{{\text{CB}}}} } \right) + {\text{CuO }}\left( {{\text{h}}_{{{\text{VB}}}}^{ + } } \right)$$

5 $${\text{CuO }}\left( {{\text{h}}^{ + }_{{{\text{VB}}}} } \right) + {\text{H}}_{2} {\text{O }} \to^{ \cdot } {\text{OH}} + H^{ + }$$

6 $${\text{CuO }}\left( {{\text{e}}^{ - }_{{{\text{CB}}}} } \right) + {\text{O}}_{2} \to^{ \cdot } {\text{O}}_{2}^{ - }$$

7 $$^{ \cdot } {\text{O}}_{2}^{ - } + 2{\text{H}}^{ + } + {\text{ e}}^{ - } \to {\text{ H}}_{2} {\text{O}}_{2}$$

8 $${\text{H}}_{2} {\text{O}}_{2} + {\text{H}}^{ + } + {\text{e}}^{ - } \to^{ \cdot } {\text{OH}} + {\text{H}}_{2} {\text{O}}$$

9 $${\text{H}}_{2} {\text{O}}_{2} + {\text{O}}_{2}^{ - } \to {\text{OH}}^{ - } +^{ \cdot } {\text{OH}} + {\text{O}}_{2}$$

Mechanism 2b of photocatalytic degradation of methyl orange dye.

**Mechanism 2a:**
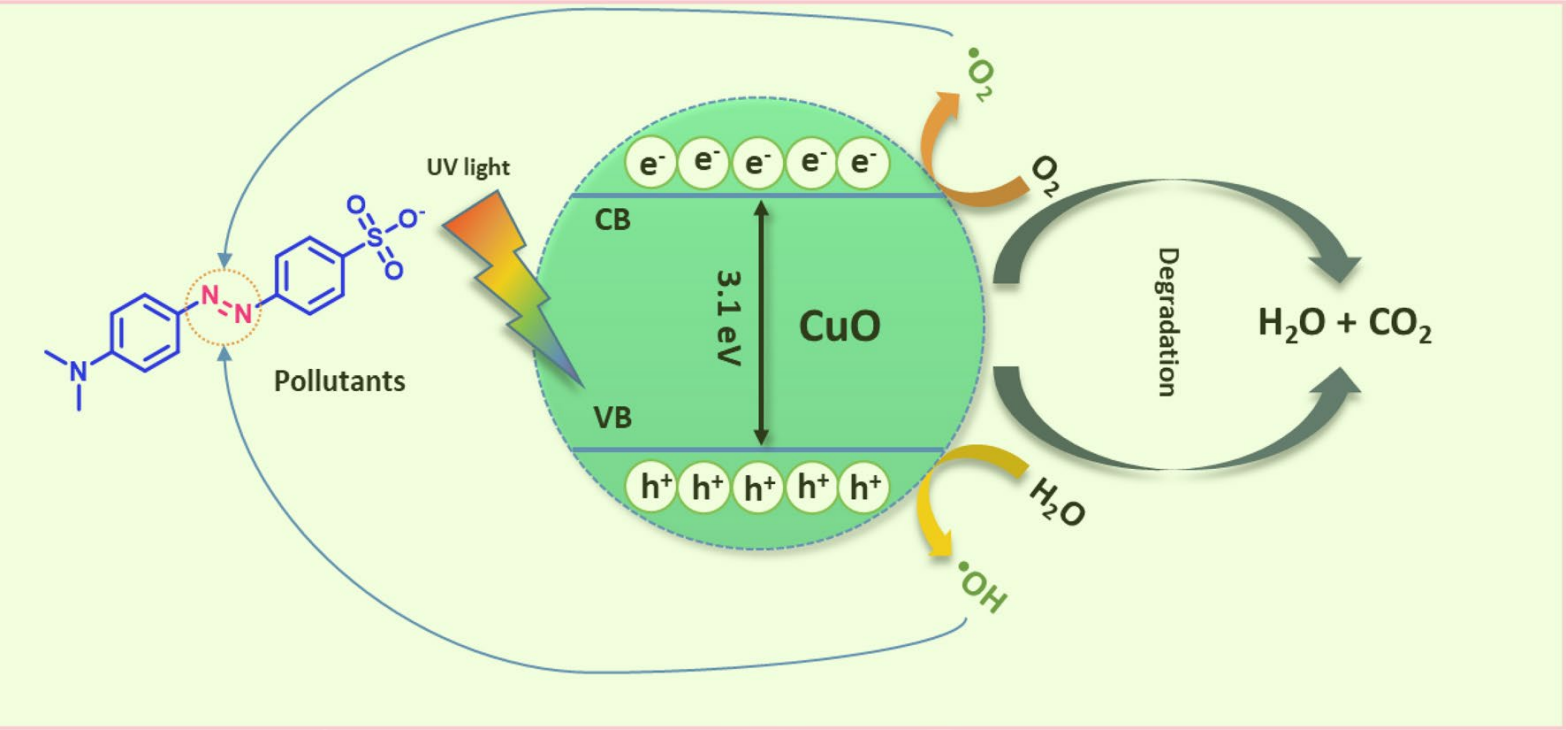
Mechanism of photocatalytic degradation of methyl orange dye.

**Mechanism 3:**
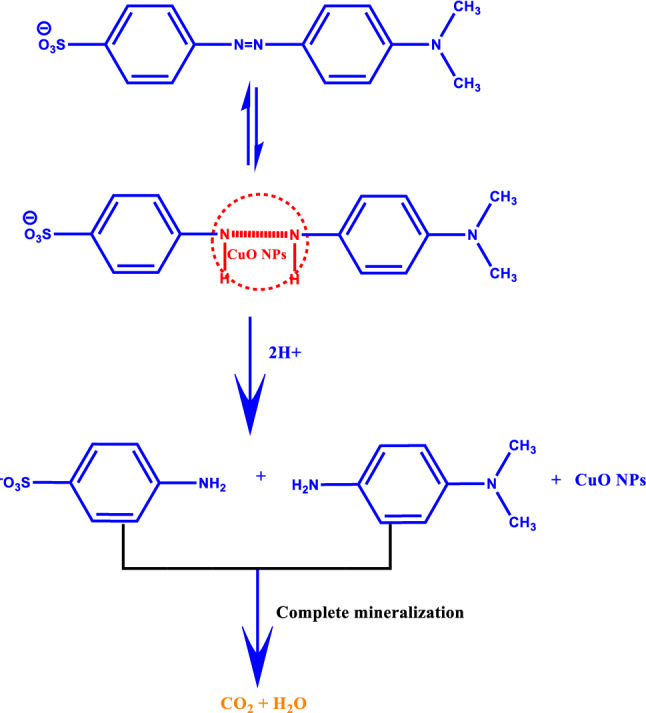
A plausible mechanism of dye degradation of MO by synthesized CuO NPs.

**Table 1:** Comparison of synthesized CuO NPs for photocatalyst application.

S. no.	Photocatalyst	Additive amount	MO concentration	Degradation rate (%)	Irradiation time	References
1	CuO/ZnO	50 mg	10 ppm	89	120 min	^57^
2	CuO	0.2 g	100 µM/100 mL	70	240 min	^34^
3	MWCNT/CuO	100 mg	50 mg/100 ml	92	6 Hrs	^58^
4	CuO/Cu_2_O–ZnO	25 mg	10 ppm	97	45 min	^32^
5	CuO	100 mg	10 mg/L	84%	120 min	This work

**Table 2:** The photodegradation efficiency of MO solutions on CuO nanoparticles obtained from different reaction conditions.

Sample	Light	Dye	Decolorization efficiency (%)			Time (min)
			Blank	Dark	photocatalysis	
CuO NPs	UV	MO	6.5	7.2	84.23	120

### CuO NPs green electrode for energy storage:

**Figure 8:**
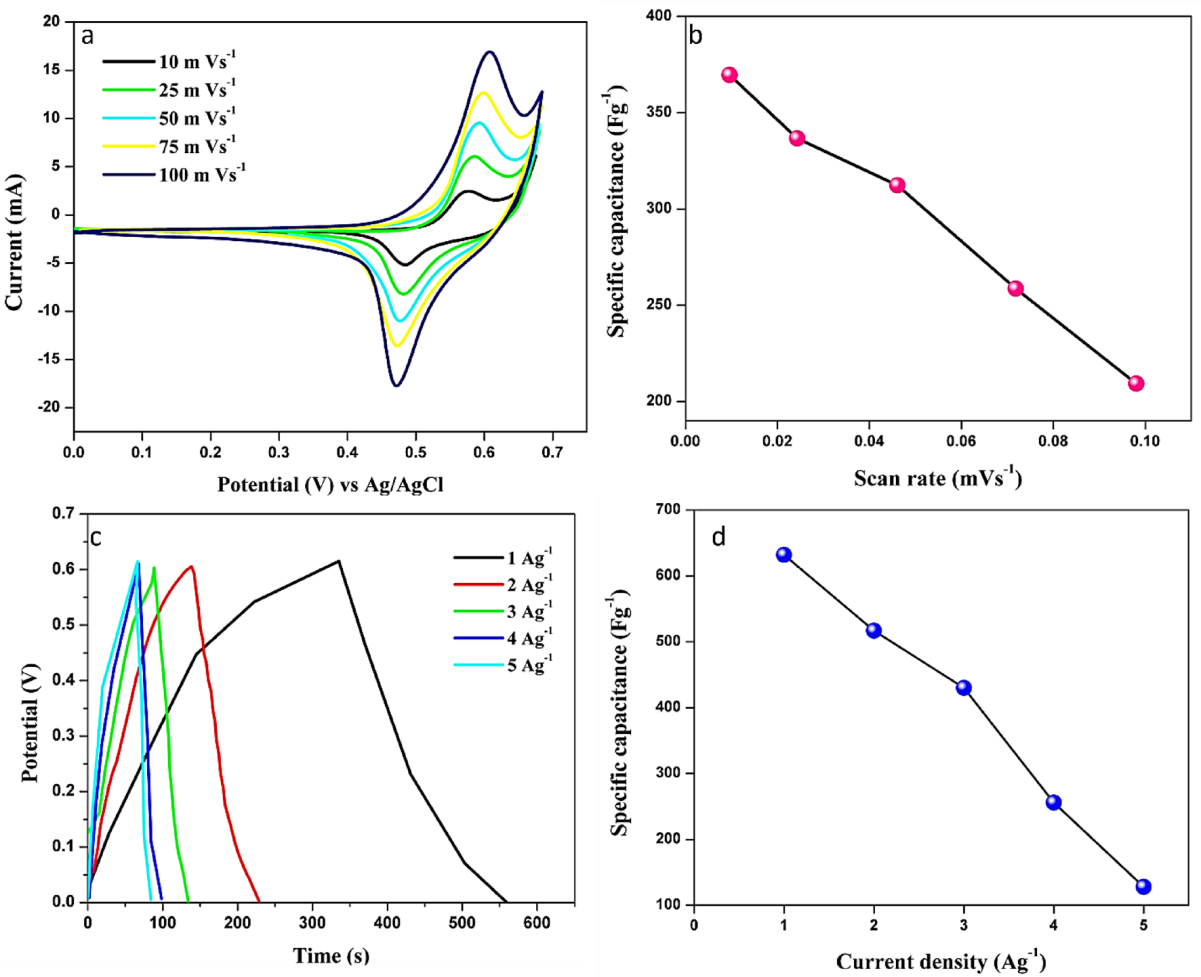
The electrochemical response of synthesized CuO NPs (**a**) CV response at various scan rates (**b**) corresponding calculated specific capacitance with respect to different scan rates and (**c**) GCD response of CuO NPS at various current densities and (**d**) calculated respective specific capacitance at different current density.

The electrochemical test was tested in a 3 M KOH as an electrolyte solution by a traditional 3-electrode system. Figure 8a shows the cyclic voltammetry of Ni foam loaded with 1 mg of biosynthesized CuO NPs modified-electrode at various scan rate ranges from 10 to 100 mV s^−1^ in the potential range of 0–0.65 V. The gradually increasing current value is noted while increasing the scan rate (Fig. 8b). The permanent faradic redox current responses were noticed generally Ni foam has a low current response compared to CuO NPs loaded Ni foam. The CuO NPs exhibited the largest integral area under the cyclic voltammetry curve and the highest current density in both anodic and cathodic peaks. The GCD was also examined in a three-electrode system using the same procedure described above. Figure 8c displays the recorded GCD curves of CuO NPs at 1 to 5 A/g. By reducing the current density, the Charge/discharge time is stabilized. The Specific capacitance values were calculated from the CV and found to be 369, 336, 312, 258, 209 Fg^−1^ for CuO NPs electrode at the scan rate of 10, 25, 50, 75, 100 mVs^−1^, and from the GCD found to be 632, 517, 430, 256 and 128 Fg^-1^ for current densities of 1,2,3,4,5 Ag^−1^ (Fig. 8d). Similarly, Ikhiya et al.^29^, synthesized similar CuO NPs using the moringa plant, and under the same electrochemical condition they achieved 338 F/g. In contrast, the Alturki ^32^ team synthesized CuO–AC (activated charcoal) from sugarcane molasses, and they attained only 245 F/g with 99% retention time, similarly, *Ravichandran* ^31^ group also reported CuO NPs with r-GO showed only 82.1 F/g of specific capacitance with 98% cyclic stability. We achieved 369 F/g of specific capacitance. As discussed earlier the heterogenous advanced material, copper composites such as CuFeS_2_^33^ and CNT@TFcP/Cu ^59^ were synthesized for better capacitance. The recently reported CuO NPs and composites were compared with our results (Table 3). Our results shows better efficient capacitance than others.

**Table 3:** Comparison for biosynthesized CuO NPs for supercapacitor applications.

S. No.	Electrode material	Sc (F/g)	Capacitance retention (%)	Electrolyte	Power density (W/Kg)	Energy density (W/Kg)	Reference
1	CuO	338	–	KOH	–	–	^27^
2	CuO	2.14	–	NaHCO_3_		0.171	^28^
3	CuO–GO	82.1	98	KOH	–	–	^29^
4	CuONPs/AC	245	99.5	Na_2_SO_4_	2091.6	45.9	^30^
5	CuFeS_2_	501	82	Na_2_SO_4_	–	–	^31^
6	Cu_2_O	87	74	Na_2_SO_4_	737	39.3	^60^
7	CNT@TFcP/Cu	280	80	H_2_SO_4_			^59^
8	CuO–rGO	137	–	Na_2_SO_4_	12 K	14	^61^
9	Cu_2_O/RGO	195	79	KOH	4114	37.7	^62^
10	CuO	369	96.4	KOH	–	–	This work

**Figure 9:**
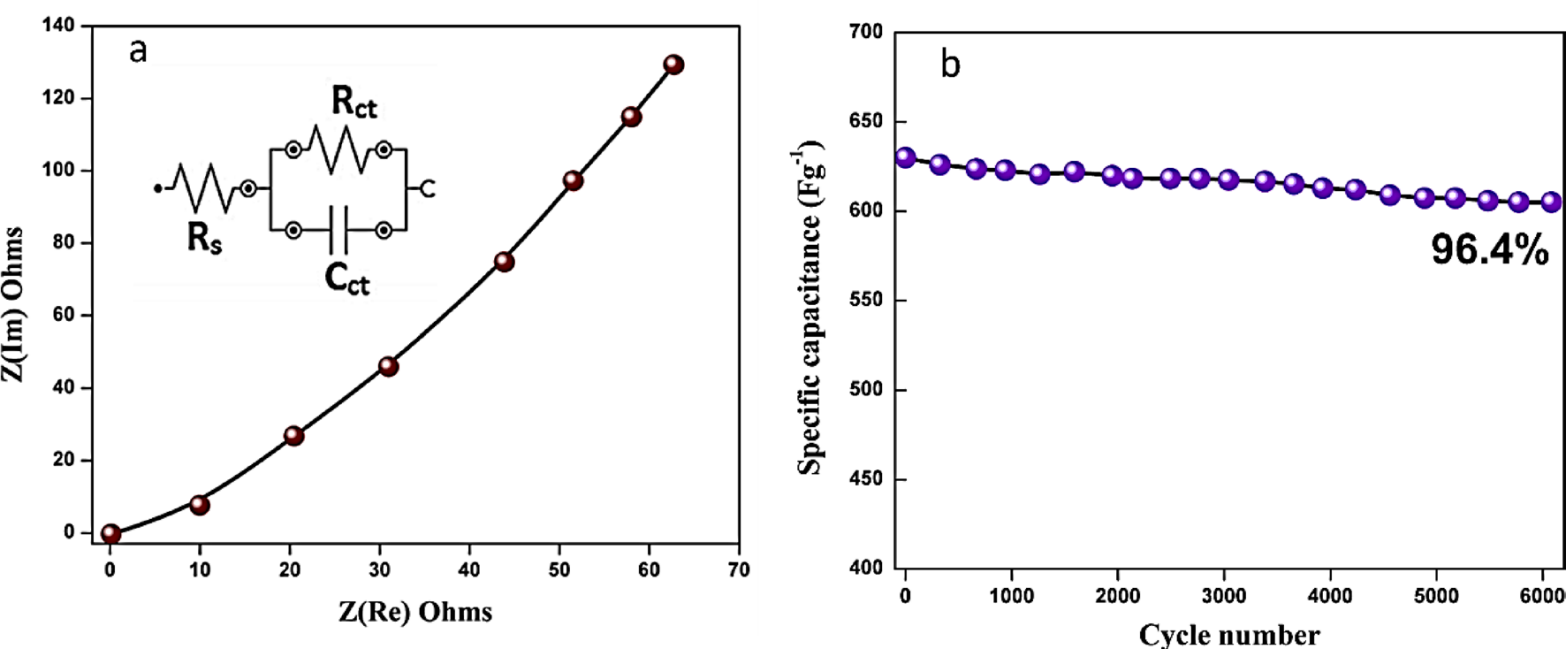
(**a**) EIS analysis of synthesized CuO NPs and (**b**) the cyclic stability.

The cycle stability of the prepared electrode plays an important role in energy applications. The prepared electrode was subjected to 6000 cycles of charge/discharge at a current density of 1 A/g and maintained 96.4% of its capacity retention (Fig. 9b). Furthermore, the EIS was tested in a high-frequency field, An EIS is recorded at a potential of 20 mV in 1 M KOH electrolyte between 100 kHz and 0.01 Hz. (Fig. 9a) Solution resistance (R_s_) and Charge transfer resistance (R_ct_) were found to be 2.62 and 14.32 Ω^.^ Having a lower value for Rs indicates the material is electrically conductive and that the active material is well attached to the current collector. Due to the high conductivity of the electrode during the reaction with electrolytes, the Nyquist plot does not show a semicircle in the high-frequency region or has a smaller R_ct_ value as shown in Fig. 8a.

The plausible redox mechanism of CuO is presented below^63^:

$${\text{CuO}} + {\text{OH}}^{ - } \leftrightarrow {\text{CuO}} + 1/2{\text{H}}_{2} {\text{O}} + {\text{e}}^{ - }$$

$${\text{CuO}} + 1/2{\text{H}}_{2} {\text{O}} + {\text{OH}}^{ - } \leftrightarrow {\text{Cu}}\left( {{\text{OH}}} \right)_{2} + {\text{e}}^{ - }$$

$${\text{CuOH}} + {\text{OH}}^{ - } \leftrightarrow {\text{CuO}} + {\text{H}}_{2} {\text{O}} + {\text{e}}^{ - }$$

$${\text{CuOH}} + {\text{OH}}^{ - } \leftrightarrow {\text{Cu}}\left( {{\text{OH}}} \right)_{2} + {\text{e}}^{ - }$$

## Conclusion

In conclusion, the greener method was used to fabricate CuO NPs using *T. terrestris* seed natural extract with an average size of 58 nm. The main novelty of this work is to satisfy the important parameters of ecological material (greener material) for dual roles such as photocatalysts and green electrodes for energy storage applications with good results. The synthesized CuO NPS was used to serve as electrode materials in a supercapacitor. The CuO nanoparticles stand out among the prepared samples with the highest specific capacitance of 632 F/g at a current density of 1 A/g, a sizable rate of capacitance, and outstanding cyclic stability (96.4 percent after 6000 rounds). CuO nanomaterial has a high electrochemical efficiency due to its ability to provide more active sites, enhance electrical conductivity, and carry out charge transfer effectively. Recent experiments have shown that CuO is capable of destroying up to 84% of methyl orange dye within 120 min. These results suggest that CuO nanomaterial can be a valuable component in energy storage devices, such as supercapacitors. Furtherly, the synthesized CuO NPs degrade the organic pollutant of MO as well as the degradation of methyl orange dye by 84% within 120 min and the outcomes demonstrated the nanomaterial's effective breakdown of contaminants, highlighting their potential for environmental rehabilitation.

Our research has conclusively demonstrated that copper oxide synthesis through green methods is a highly effective and cost-efficient solution for pollutant adsorption. This innovative approach also has tremendous potential for wastewater treatment and energy storage in future energy devices. Our findings are a testament to the serious, talented, and novel research that we have conducted shows that synthesized copper oxide through green methods is a highly suitable solution for pollution control and energy storage devices.

## Data Availability

The datasets used and/or analyzed during the current study are available from the corresponding author on reasonable request.
